# Detecting a Cortical Fingerprint of Parkinson's Disease for Closed-Loop Neuromodulation

**DOI:** 10.3389/fnins.2016.00110

**Published:** 2016-03-30

**Authors:** Kevin Kern, Georgios Naros, Christoph Braun, Daniel Weiss, Alireza Gharabaghi

**Affiliations:** ^1^Division of Functional and Restorative Neurosurgery and Centre for Integrative Neuroscience, Eberhard Karls University TuebingenTuebingen, Germany; ^2^Magnetoencephalography Center, Eberhard Karls University TuebingenTuebingen, Germany; ^3^Center for Mind/Brain Sciences (CIMeC), University of TrentoItaly; ^4^Department for Neurodegenerative Diseases and Hertie Institute for Clinical Brain Research and German Centre of Neurodegenerative Diseases (DZNE), Eberhard Karls University TuebingenTuebingen, Germany

**Keywords:** deep brain stimulation, Parkinson's disease, beta cortico-muscular coherence, source space, neurophysiological biomarker, cortical targeting, closed-loop stimulation

## Abstract

Recent evidence suggests that deep brain stimulation (DBS) of the subthalamic nucleus (STN) in Parkinson's disease (PD) mediates its clinical effects by modulating cortical oscillatory activity, presumably via a direct cortico-subthalamic connection. This observation might pave the way for novel closed-loop approaches comprising a cortical sensor. Enhanced beta oscillations (13-35 Hz) have been linked to the pathophysiology of PD and may serve as such a candidate marker to localize a cortical area reliably modulated by DBS. However, beta-oscillations are widely distributed over the cortical surface, necessitating an additional signal source for spotting the cortical area linked to the pathologically synchronized cortico-subcortical motor network. In this context, both cortico-subthalamic coherence and cortico-muscular coherence (CMC) have been studied in PD patients. Whereas, the former requires invasive recordings, the latter allows for non-invasive detection, but displays a rather distributed cortical synchronization pattern in motor tasks. This distributed cortical representation may conflict with the goal of detecting a cortical localization with robust biomarker properties which is detectable on a single subject basis. We propose that this limitation could be overcome when recording CMC at rest. We hypothesized that—unlike healthy subjects—PD would show CMC at rest owing to the enhanced beta oscillations observed in PD. By performing source space analysis of beta CMC recorded during resting-state magnetoencephalography, we provide preliminary evidence in one patient for a cortical *hot spot* that is modulated most strongly by subthalamic DBS. Such a spot would provide a prominent target region either for direct neuromodulation or for placing a potential sensor in closed-loop DBS approaches, a proposal that requires investigation in a larger cohort of PD patients.

## Introduction

Cortico-subcortical networks of Parkinson's disease (PD) patients are characterized by pathological circuit dynamics such as dysfunctional synchronization in the beta-frequency (12–35 Hz) band (Little and Brown, 2014). Dopaminergic medication and electrical stimulation may rebalance and clinically improve these altered interactions both locally and on a network level (Kühn et al., 2008; Litvak et al., 2011; Eusebio et al., 2012; Hirschmann et al., 2013b; Weiss et al., 2015). Even in PD patients with early motor complications, deep brain stimulation (DBS) of the subthalamic nucleus (STN) provides better relief (alleviating motor symptoms and improving life quality) than medication (Schuepbach et al., 2013).

Two lines of evidence nominate the cortex as a target for modulating the dysfunctional network dynamics in PD: In patients, cortical beta activity precedes pathological subcortical synchronization (Marreiros et al., 2013). Furthermore, findings in both animal models of DBS (Gradinaru et al., 2009; Li et al., 2012) and patients (de Hemptinne et al., 2015) suggest that anti-dromically activated responses in the motor cortex are involved in the beneficial effects of STN DBS. Although previous studies of electrical cortical stimulation (CS) in PD revealed lower efficacy compared to DBS (Cilia et al., 2007; Moro et al., 2011; Bentivoglio et al., 2012), recent technological developments of implantable devices for simultaneously sensing and stimulating (Afshar et al., 2012) have refueled the interest in CS by applying closed-loop devices (Rosin et al., 2011; Beuter et al., 2014). Simultaneous magnetoencephalography (MEG) and local field potential (LFP) recordings in the STN of PD patients (using implanted DBS electrodes with externalized leads) detected long-range functional connectivity between STN and the ipsilateral sensorimotor and premotor cortex in the beta frequency range, suggesting the involvement of the hyperdirect pathway (Hirschmann et al., 2011; Litvak et al., 2011). More recently, electroencephalography (EEG) has also been applied to capture cortical beta-gamma phase-amplitude coupling (PAC) as a functional marker in PD while demonstrating its modulation by levodopa (Swann et al., 2015). However, the elevation of the EEG PAC signal in PD patients compared to healthy controls was not demonstrable on a single subject basis, but only at the group level (Swann et al., 2015). We therefore still lack a non-invasive technique that could be applied before surgery on a single subject basis to functionally localize a cortical target area informative on pathological beta oscillatory characteristics for either direct CS or as a sensor for closed-loop DBS.

There is converging evidence, that enhanced cortical beta activity is a prominent feature in the pathophysiology of PD (Kühn et al., 2008; Eusebio et al., 2011; Airaksinen et al., 2012; Little and Brown, 2012). Moreover, the hyperdirect pathway, connecting the STN and the motor cortical area is believed to mediate a DBS-related decrease of this exaggerated beta activity in parallel to alleviation of bradykinesia-rigidity symptoms (Kühn et al., 2006; Whitmer et al., 2012). Therefore, the most intuitive candidate marker is expected within the beta frequency range in order to detect a reliable cortical biomarker for PD. Oscillatory activity in the beta range, however, is widely distributed over the cortical surface. Additional measures are warranted to spot the cortical area reliably modulated by DBS. Cortico-muscular coherence (CMC) might provide such an additional measure. During motor tasks, CMC generally displays a spatially distributed cortical representation (Crone et al., 1998a,b; Grosse-Wentrup et al., 2011; Hipp et al., 2011), thereby conflicting with the goal of detecting a cortical hot spot. There is also a large inter-subject variability of CMC in PD patients during a motor task (Kühn, 2004; Kühn et al., 2008; Tropini et al., 2011; Weiss et al., 2012; Selzler et al., 2013) most probably explaining the variable findings in previous motor task related CMC studies in PD (Airaksinen et al., 2015).

We propose that this limitation could be overcome when performing the CMC measurement at rest. We suggest that—unlike in healthy subjects—(I) CMC would be detectable in the PD condition even in the absence of movement due to the disease-specific enhanced beta oscillations. Furthermore, we hypothesize that (II) the cortical CMC spot would topographically converge with the cortical spot modulated most strongly by subthalamic DBS. To provide proof-of-concept evidence, we implemented a MEG set-up for resting-state CMC and DBS to perform source space analysis of related cortical activity.

## Experimental set-up

### Recording

The measurements were performed inside a magnetically shielded room (Vakuumschmelze, Hanau, Germany) with a whole-head 275-channel MEG (CTF, VSM Medtech, Port Coquitlam, Canada) at a sampling rate of 1172 Hz. Recordings included bipolar electromyography (EMG) from the right flexor carpi radialis muscle (grounded to elbow), bipolar electrooculography (EOG) and electrocardiography (ECG). The patient was seated comfortably and asked to remain motionless. Her gaze was focused on a fixation cross at eye level. The pre-programmed stimulation was switched off 30 min beforehand to obtain reliable wash-out of subthalamic DBS effects (Cooper et al., 2013; Weiss et al., 2013). We recorded 6 min (360 s) of resting-state MEG without stimulation (rest). This baseline measurement was followed by recordings during 10 stimulation trials, each lasting 2 min; 1 min stimulation-on followed by 1 min stimulation-off. For data analysis, we selected the first 40 s of the stimulation-off period of each trial. This resulted in an overall time-period of ~360 s after artifact rejection. Time periods immediately following DBS were compared with the 360 s of baseline measurement at rest before the stimulation trials. This approach was chosen because: (i) methods for suppressing the stimulation artifact in MEG recordings during simultaneous monopolar DBS might not be sufficiently effective for interpreting stimulation results (Devos, 2004; Silberstein et al., 2005; Airaksinen et al., 2012). By analyzing the period immediately after stimulation, stimulation artifacts are avoided. (ii) Even short term stimulation has neuronal effects that last for several seconds or minutes after stimulation (Kühn et al., 2008; Bronte-Stewart et al., 2009; Whitmer et al., 2012). The time period immediately following stimulation might therefore provide information on stimulation-induced physiological effects. (iii) By comparing this data to the resting measurements before stimulation trials, any carry-over effects in the data used as baseline can be excluded. Source space analysis was performed using a T2-weighted individual MRI.

### Pre-processing

Notwithstanding stimulation, the DBS hardware itself induced relevant MEG artifacts. These were removed using temporal signal space separation (tSSS; Taulu and Simola, 2006; Taulu and Hari, 2009), which was applied with a subspace correlation limit of 0.9 (Medvedovsky et al., 2009; Airaksinen et al., 2011). The Dynamic Imaging of Coherent Sources (DICS) beamformer has been shown to reliably suppress metal artifacts of implanted DBS electrode leads in the stimulation-off mode as well as with externalized extension cables, i.e., an impulse generator (IPG) outside the body (Litvak et al., 2010). However, it has not yet been shown that this approach also removes the artifacts induced by an implanted IPG during breathing or even during stimulation.

By contrast, tSSS has been shown to overcome these additional artifacts when applied in an Electa Neuromag. Together with the developers of tSSS, we have therefore implemented tSSS in our CTF MEG system. The data presented here was preceded by phantom and *in vivo* studies to optimize the device settings so as to reliably suppress artifacts. When signal quality did not allow a reliable rejection of artifacts, the algorithm refused the artifact rejection *per se*. In these pre-studies, we reliably localized movement-related activity and event-related potentials to their anticipated cortical localization during stimulation (unpublished data).

The patient's magnetic resonance images (MRI) were interpolated to a resolution of 256 × 256 × 256 voxels. Fiducial (nasion, left, and right preauricular) and anatomical landmarks (anterior and posterior commissure, mid-sagittal plane) were used to align the MRI to standard space of the Montreal Neurological Institute (MNI). Finally, the source space was defined by a single shell segmented model and lead fields with a resolution of 8 mm.

We applied a first order zero-phase lag finite impulse response bandpass filter (1–40 Hz) to the MEG time series, and highpass filters of 0.5 Hz to the ECG, 0.05 Hz to the EOG, and 2 Hz to the EMG, respectively, to preserve relevant spectral components in the signals. Thereafter, we applied an independent component analysis (ICA) and correlation analysis to identify and remove eye movement, blink, and cardiac-related artifacts. We decorrelated each trial of the MEG sensor time series into 40 independent components via fast independent component analysis. Both independent components, which maximally correlated with the ECG and EOG, were labeled artifacts and skipped during back-projection of the independent components to sensor level. An additional visual inspection, where we focused on the eye movement, blink, and cardiac-related artifacts in the time series of the components, verified the respective artifacts. The magnetic fields of the single sensors were recomputed using an interpolation toward average head position throughout the recordings. All trials and conditions were divided into 5-s epochs. Finally, z-value-transformed epoch-wise artifact rejection was performed using a cutoff-value of 20.

### Data analysis

**Figure 1:**
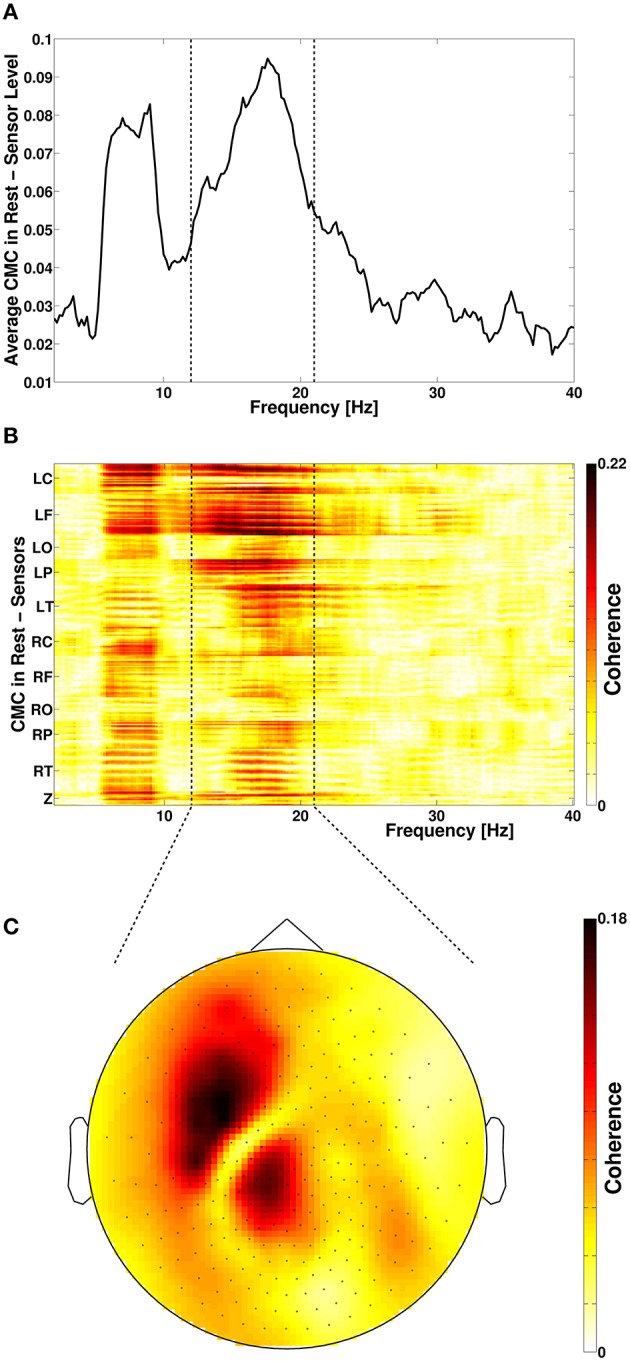
**(A)** Frequency spectrum of resting-state cortico-muscular coherence (CMC) of all sensors. Dotted vertical lines indicate the beta-frequency range which was analyzed. **(B)** Spatial distribution of CMC. **(C)** Topoplot of beta-band CMC in MEG sensor space. L, left; R, right; C, central; F, frontal; O, occipital; P, parietal; T, temporal; Z, midline.

We applied a previously described approach (Litvak et al., 2011) to identify the frequency band with maximal cortico-muscular coherence (CMC) and to calculate source space CMC. We rectified the EMG channel (Myers et al., 2003; Yao et al., 2007) and calculated cross-spectra with all MEG sensor time series from 2 to 40 Hz by tapering the 5-s epochs using slepian functions (Mitra and Pesaran, 1999) and 2 Hz spectral smoothing resulting in a frequency resolution of δ*f* = 0.2 Hz (see Figure 1A). Source space CMC was then calculated by using these cross-spectra and Dynamic Imaging of Coherent Sources (DICS) beamforming (Gross et al., 2001). Pre-processing with tSSS has previously been shown not to restrict the usage of a subsequent beamformer analysis (Hillebrand et al., 2013). Common spatial DICS filter of both conditions, i.e., rest and stimulation, were calculated beforehand and used for source space CMC calculation. Calculating a common spatial filter for beamforming is usually applied when comparing different conditions after beamforming to avoid biased results due to different spatial filters of the conditions.

For the statistical comparison of the rest and stimulation condition for both CMC and power, a non-parametric cluster-based permutation approach was used to correct for multiple comparisons (Maris and Oostenveld, 2007; Oostenveld et al., 2011). We computed voxel-wise *t*-values between the rest and stimulation condition and clustered voxels exceeding *p* < 0.001 (uncorrected). The sum of the *t*-value within the clustered voxel area defined the cluster-level statistics. By randomly permuting the data between rest and stimulation condition for 1000 times, we obtained a reference null distribution of the maximum cluster-level statistics. In order to correct for multiple comparisons, the maximum cluster-level statistics that differed from the reference null distribution with *p* < 0.05 (corrected) were considered significant. All data analysis was performed offline with custom written scripts and FieldTrip toolbox (Oostenveld et al., 2011) in MATLAB® (R2011a, The MathWorks® Inc.,).

## Empirical data

An idiopathic akinetic-rigid PD patient (female; age: 69 years; disease duration: 12 years; UPDRS III (motor part) stimulation on/off: 15/20) participated after giving written informed consent. We examined the patient 5 months after DBS surgery with bilateral electrode implantation in the STN (Medtronic 3389 leads and Activa PC® stimulator, Minneapolis, USA). The physiological effects of unilateral DBS (left hemisphere) were studied using the same stimulation parameters as applied clinically (monopolar, contact 2-G+, 1.5 V, 60 μs, 125 Hz) while the patient was in medication-on (levodopa equivalent dose = 581 mg; Deuschl et al., 2006). The study was conducted with the patients' informed consent and in accordance with the guidelines approved by the local ethics committee of the University Hospital Tuebingen.

**Figure 2:**
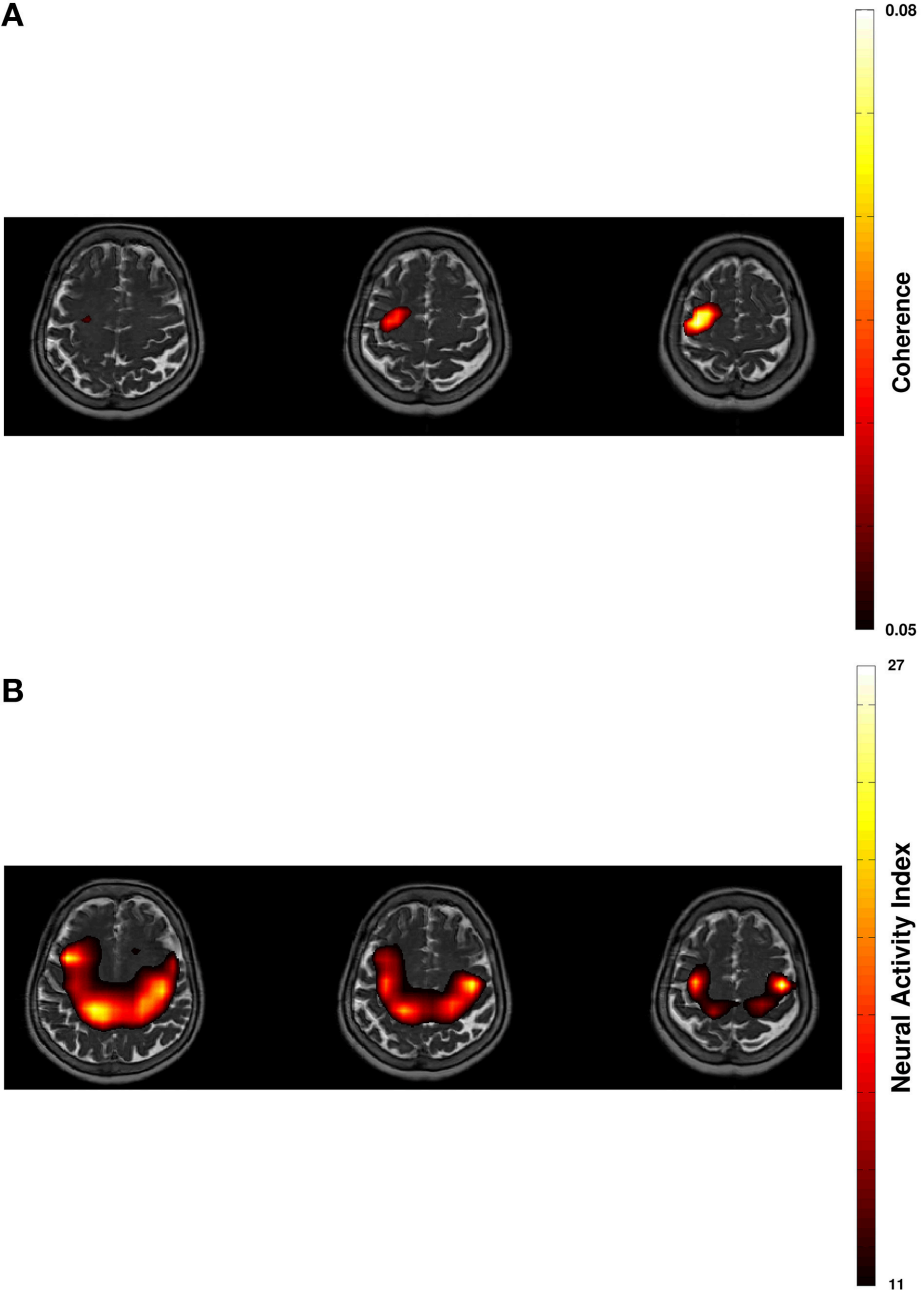
**Projection of beta-band cortico-muscular coherence (A) and of cortical beta power (B) in resting-state on transversal slices of individual T2-weighted MRI with 60% thresholding**.

**Figure 3:**
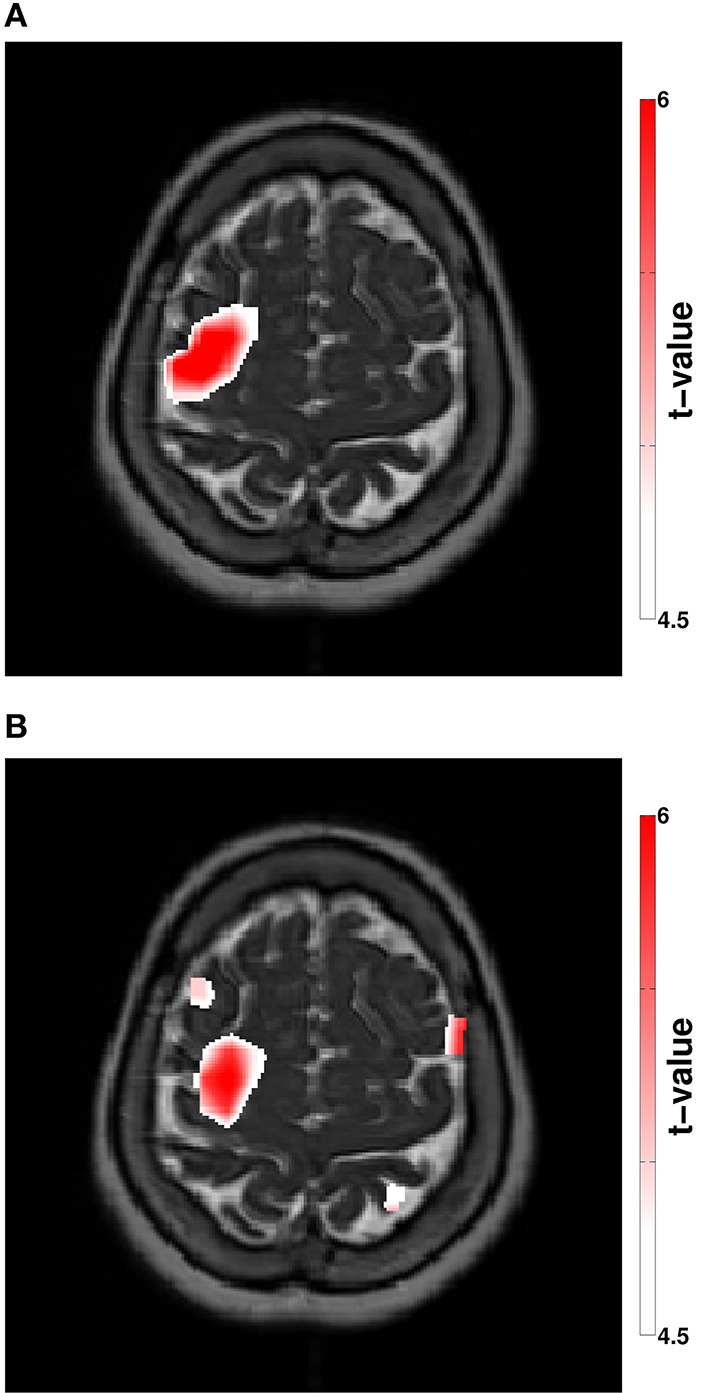
**(A)** Stimulation modulated beta-CMC showing the difference between the rest and the stimulation condition. **(B)** Stimulation modulated cortical beta power showing the difference between the rest and the stimulation condition. The color bar indicates the *t*-values according to a non-parametric cluster-based permutation approach (*p* < 0.05, corrected).

Spectral analysis of CMC at baseline, i.e., in rest and stimulation-off, revealed that the strongest connectivity occurred in the beta-band (Figure 1A). Scanning all sensors showed predominant involvement of the hemisphere contralateral to the EMG recording (Figure 1B) with a topography suggestive of one major source in MEG sensor space (Figure 1C). Source space CMC analysis showed the maximum of the beta-CMC within the motor cortex of the hemisphere contralateral to the muscle (Figure 2A) as opposed to a distributed pattern of cortical activation in the beta-band (Figure 2B). During STN-DBS, the same beta-CMC (Figure 3A) and beta power (Figure 3B) source-cluster revealed the strongest stimulation-induced changes, i.e., decreases of CMC and cortical power in the beta-band.

## Discussion

We performed source space analysis of beta CMC recorded during resting-state MEG and DBS and provide proof-of-concept evidence for a cortical *hot spot* that is most strongly modulated by subthalamic DBS. Since this study is carried out in only one subject, this proposal requires investigation in a larger cohort of PD patients to probe the robustness of this approach, e.g., for different disease states.

The present work suggests that a circumscribed cortical area involved in the pathologically synchronized cortico-subcortical-muscular motor network can be detected non-invasively in PD on single subject level, which constitutes a mandatory prerequisite for future closed-loop neurostimulation strategies on the basis of a cortical sensor. Due to its high spatial resolution of ~2 mm (Pizzella and Romani, 1990), MEG is as an excellent tool for non-invasive mapping prior to neurosurgical procedures. Up to date, MEG examinations have been successfully used for presurgical evaluation of epilepsy patients (Knowlton and Shih, 2004) and patients with tumors in eloquent areas (Rezai et al., 1996; Orrison, 1999). Integration of the MEG information in the stereotactic planning software has been used for electrode placement (Agirre-Arrizubieta et al., 2014) and to aid safe resection of tumors (Kelly, 1996; Rezai et al., 1996; Orrison, 1999). The knowledge acquired with the presented approach may thus be integrated in stereotactic planning for the placement of cortical implants for direct neuromodulation or for placing a potential sensor in closed-loop DBS approaches. We did not intend to describe a specific extent or location of such a cortical area, since these parameters may vary between patients. However, in accordance with previous literature on the hyperdirect cortico-subthalamic pathway recorded with cortico-subthalamic coherence (Hirschmann et al., 2011), the data presented here suggests that this cortical area covers a part of the medial motor und premotor cortex.

The involvement of the cortical areas associated with motor processing in Parkinson tremor is well-documented (Timmermann et al., 2003; Hirschmann et al., 2013a). However, source space topographic information about CMC in akinetic-rigid PD and in the absence of movement is rather sparse. As a conceptual novelty of this work, we captured the cortical spot of pathological resting-state CMC in the beta band. An implantable cortical sensor for closed-loop applications needs to be spatially restricted from a neurosurgical point of view. Therefore, the cortical target point would be defined as the area which addresses both networks, i.e., the rigidity/bradykinesia and the tremor network. The cortical area involved most strongly in the rigidity/bradykinesia network observed here seems to be more restricted than the tremor network reported by Timmermann and colleagues. Since still being part of the latter such an area would qualify as the common hotspot.

Our concept suggests capturing a cross spectrum for every patient to detect the individual peak frequency band of CMC (see Figure 1A). As expected, the presented case also showed the peak CMC in the beta-band followed by the alpha-band. This observation is in accordance with a large body of literature reporting relating enhanced beta oscillations both in the STN and on the cortical level to the pathophysiology of PD (Kühn et al., 2008; Eusebio et al., 2011; Airaksinen et al., 2012; Little and Brown, 2012; Whitmer et al., 2012). Moreover, beta-band oscillations have repeatedly been shown to mediate cortico-muscular communication (Salenius et al., 2002; Baker, 2007; Engel and Fries, 2010; Weiss et al., 2012; Airaksinen et al., 2015). In contrast to beta-CMC, alpha-CMC is not consistently found (Kilner et al., 2000; Budini et al., 2014) and may be confounded by coherence occuring at double tremor frequency (Timmermann et al., 2003). Therefore, it is not surprising that we did not find any significant source cluster for alpha-CMC in the resting state and in the absence of tremor.

STN stimulation is known to modulate spontaneous activity and somatosensory evoked responses over the sensorimotor cortex (Mäkelä et al., 2007; Airaksinen et al., 2011). Furthermore, excessive beta oscillatory activity is suppressed by STN-DBS in PD along with symptom alleviation (Kühn et al., 2008; Whitmer et al., 2012; Little and Brown, 2014). This correlation of therapeutic beta activity modulation and motor symptom alleviation is not surprising, given that oscillatory beta activity relates to motor processing, sensorimotor control, and cortico-peripheral interactions (Brovelli et al., 2004; Schoffelen et al., 2005; Baker, 2007; Chakarov et al., 2009; Engel and Fries, 2010). Cortical beta band activity may parallel the maintenance of the sensorimotor state (status quo; Engel and Fries, 2010). Therefore, decreased motor cortical beta power, as found in this study, may represent a release of cognitive resources, which restores the ability of motor self-control mediated by cortico-basal ganglia-thalamo-cortical loops (Kringelbach et al., 2010; McIntyre and Hahn, 2010; Little and Brown, 2012). As already described previously (Whitmer et al., 2012; Weiss et al., 2015), STN stimulation suppresses cortical beta band activity in motor-related areas. Thus, the findings reported here are in line with these previous results.

Cortico-peripheral interactions as measured by CMC in the beta frequency band may be increased by levodopa administration (Salenius et al., 2002). The effect of STN stimulation was more variable showing a slight beta band CMC increase on fine-motor integration (Weiss et al., 2012) and variable outcomes during joint movement (Airaksinen et al., 2015). This case study suggests that, in the resting state, this long-distance corticomuscular synchronization is decreased by STN-DBS. This tallies with the anticipated dysbalanced drive from cortex to muscle in PD (Salenius et al., 2002; Hirschmann et al., 2013b). The concept of maintaining the status quo via sensorimotor activity in beta band may therefore be extended to beta band CMC as well (Baker, 2007; Engel and Fries, 2010). Decreasing beta CMC in the resting state through STN-DBS may thus represent a reduction of an abnormal persistence of the status quo, i.e., of the pathological oscillatory drive to the muscle in PD (Brown et al., 2001; Marsden et al., 2001). It should be noted, however, that CMC in itself may have a functional role in the motor system apart from the cortical oscillatory activity (Baker and Baker, 2003), thus deserving a consideration independent from cortical beta band power modulation.

However, previous work performing resting-state MEG recordings in PD patients with DBS has neither captured CMC nor has it analyzed source level oscillatory activity (Cao et al., 2015). Only movement-related beta-CMC is currently being investigated as a physiological marker in PD patients undergoing DBS surgery (Hirschmann et al., 2011; Airaksinen et al., 2015). Although, these studies focused on the impact of levodopa medication and DBS on CMC as physiological predictors of clinical outcome, an application of this technique for delineating a cortical *hot spot* for surface stimulation remains elusive. Airaksinen and co-workers suggested that the cortical representation of beta-CMC and the cortical area modulated by DBS might even differ (Airaksinen et al., 2015). Based on our findings, we suggest that the cortical representation of beta-CMC during resting state might be more restricted.

We therefore suggest that resting-state CMC may eliminate the large inter-individual variability inherent to movements in PD patients in general (Kühn, 2004; Kühn et al., 2008; Tropini et al., 2011; Selzler et al., 2013) and of the related CMC in particular as observed by Airaksinen et al. (2015). Moreover, we demonstrate that source reconstruction—and co-location—of both CMC (Figure 3A) and oscillatory power (Figure 3B) during DBS reveals the cortical convergence of pathological synchronization within a cortical hot spot.

Using simultaneous MEG and LFP recordings, motor cortical areas directly connecting STN and cortex, i.e., mediated via the hyperdirect pathway, were identified in source space thereby disentangling different sub-frequency bands within the beta-band and attributing the upper beta-band to cortico-subcortical interactions (Hirschmann et al., 2011). In the present study, we did not capture cortico-subcortical coherence, which would have necessitated externalized leads. Future studies will therefore need to explore frequency-specific interactions between local beta activity and rather long-range interaction with the sensorimotor loop, i.e., for STN/cortex, muscles/cortex, and STN/muscles. Corroborating results for a direct connection between STN and cortex were however obtained by quantitative modeling of axonal fiber activation (Hartmann et al., 2015) or a combination of diffusion tensor imaging (DTI) and electrocorticographic (ECoG) electrodes (Whitmer et al., 2012).

More recently, ECoG (de Hemptinne et al., 2015) and EEG (Swann et al., 2015) have also been applied to capture cortical beta-gamma phase-amplitude coupling (PAC) as a functional marker, while demonstrating its modulation by DBS (de Hemptinne et al., 2015) and levodopa (Swann et al., 2015); PAC might provide an even more robust biomarker than beta oscillations in PD patients (de Hemptinne et al., 2015; Swann et al., 2015). Capturing PAC in MEG source space, however, poses methodological challenges. We are therefore currently applying EEG to compare different physiological markers in PD patients, e.g., for determining topographic and functional differences between PAC and beta oscillations. Future physiological studies will moreover need to compare the interactions in medication on/off and to capture clinical lateralization scores as well. Medication may modulate the very same networks that are modulated by DBS, and the observed DBS effects might even be more pronounced in the medication off state.

This proof-of-concept is however the first to show that beta-CMC delineates the very same cortical area as modulated by STN-DBS, and is meant to inspire and encourage other groups to challenge this testable hypothesis by examining their patients with resting-state CMC as well. These future larger scale studies will then have to address further questions, such as (1) can resting-state CMC be detected in all PD patients, (2) is resting-state CMC correlated with the severity of the disease, (3) does the cortical hot spot vary between patients, and (4) do peak frequencies vary between patients?

## Conclusion

We argue that the particular pathophysiology, i.e., the increased synchronization in the oscillatory beta-frequency band, in advanced Parkinson's disease (PD) would facilitate the non-invasive detection of a cortical *hot spot* since cortico-muscular communication is also mediated in the very same frequency band.

We demonstrated that both clusters projected to the same anatomically plausible area in the primary motor/ premotor area and that these overlapping source clusters revealed the strongest stimulation induced changes for CMC (Figure 3A) and cortical power (Figure 3B). The decreases of CMC and cortical power were both in the same beta-band. Only a circumscribed area of the whole extended region with increased cortical beta activity (Figure 2B) was significantly reduced by DBS (Figure 3B). All these complementary and consistent findings cannot be explained by random collocation and are therefore not compromised by the sample size of one. However, the inherent intersubject variability in signals will necessitate the study of further patients to draw definite conclusions.

The novelty of this concept is grounded in the new combination of methods rather than in a new methodology, i.e., by combining resting-state (instead of the usual movement related) cortico-muscular coherence (CMC) with source space analysis of magnetencephalography recordings. Future studies will need to reveal which cortical recording technique, MEG or EEG, is better suited to detect the cortical *hot spot* before surgery.

The underlying hypotheses are (a) that resting-state CMC would be detectable in PD (other than in healthy subjects), (b) that resting-state CMC would avoid the known variability of movement related CMC in PD, and (c) that pathologically synchronized loops would converge on the cortical level. Such a topographic convergence within a cortical-subcortical-muscular network would provide a prominent target region either for direct neuromodulation or for placing a potential *sensor* in closed-loop DBS approaches since DBS was recently shown to mediate its therapeutic effects also via remote cortical modulation.

## Author contributions

KK designed and performed research, analyzed data, and wrote the paper. GN designed and performed research. CB designed research and edited the paper. DW designed research and edited the paper. AG designed research, analyzed data, and wrote the paper.

### Conflict of interest statement

The authors declare that the research was conducted in the absence of any commercial or financial relationships that could be construed as a potential conflict of interest.

## References

[B1] Afshar P. Khambhati A. Stanslaski S. Carlson D. Jensen R. Linde D.. (2012). A translational platform for prototyping closed-loop neuromodulation systems. Front. Neural Circuits 6:117. 10.3389/fncir.2012.00117 23346048 PMC3551193

[B2] Agirre-Arrizubieta Z. Thai N. J. Valentín A. Furlong P. L. Seri S. Selway R. P.. (2014). The value of Magnetoencephalography to guide electrode implantation in epilepsy. Brain Topogr. 27, 97–207. 10.1007/s10548-013-0330-x 24249204

[B3] Airaksinen K. Butorina A. Pekkonen E. Nurminen J. Taulu S. Ahonen A.. (2012). Somatomotor mu rhythm amplitude correlates with rigidity during deep brain stimulation in Parkinsonian patients. Clin. Neurophysiol. Off. J. Int. Fed. Clin. Neurophysiol. 123, 2010–2017. 10.1016/j.clinph.2012.03.004 22513261

[B4] Airaksinen K. Mäkelä J. P. Nurminen J. Luoma J. Taulu S. Ahonen A.. (2015). Cortico-muscular coherence in advanced Parkinson's disease with deep brain stimulation. Clin. Neurophysiol. 126, 748–755. 10.1016/j.clinph.2014.07.025 25218364

[B5] Airaksinen K. Mäkelä J. P. Taulu S. Ahonen A. Nurminen J. Schnitzler A.. (2011). Effects of DBS on auditory and somatosensory processing in Parkinson's disease. Hum. Brain Mapp. 32, 1091–1099. 10.1002/hbm.21096 20645306 PMC6870287

[B6] Baker M. R. Baker S. N. (2003). The effect of diazepam on motor cortical oscillations and corticomuscular coherence studied in man. J. Physiol. (Lond). 546, 931–942. 10.1113/jphysiol.2002.029553 12563016 PMC2342588

[B7] Baker S. N. (2007). Oscillatory interactions between sensorimotor cortex and the periphery. Curr. Opin. Neurobiol. 17, 649–655. 10.1016/j.conb.2008.01.007 18339546 PMC2428102

[B8] Bentivoglio A. R. Fasano A. Piano C. Soleti F. Daniele A. Zinno M.. (2012). Unilateral extradural motor cortex stimulation is safe and improves Parkinson disease at 1 year. Neurosurgery 71, 815–825. 10.1227/NEU.0b013e318266e6a5 22791032

[B9] Beuter A. Lefaucheur J.-P. Modolo J. (2014). Closed-loop cortical neuromodulation in Parkinson's disease: an alternative to deep brain stimulation? Clin. Neurophysiol. Off. J. Int. Fed. Clin. Neurophysiol. 125, 874–885. 10.1016/j.clinph.2014.01.006 24555921

[B10] Bronte-Stewart H. Barberini C. Koop M. M. Hill B. C. Henderson J. M. Wingeier B. (2009). The STN beta-band profile in Parkinson's disease is stationary and shows prolonged attenuation after deep brain stimulation. Exp. Neurol. 215, 20–28. 10.1016/j.expneurol.2008.09.008 18929561

[B11] Brovelli A. Ding M. Ledberg A. Chen Y. Nakamura R. Bressler S. L. (2004). Beta oscillations in a large-scale sensorimotor cortical network: directional influences revealed by Granger causality. Proc. Natl. Acad. Sci. U.S.A. 101, 9849–9854. 10.1073/pnas.0308538101 15210971 PMC470781

[B12] Brown P. Marsden J. Defebvre L. Cassim F. Mazzone P. Oliviero A.. (2001). Intermuscular coherence in Parkinson's disease: relationship to bradykinesia. Neuroreport 12, 2577–2581. 10.1097/00001756-200108080-00057 11496152

[B13] Budini F. McManus L. M. Berchicci M. Menotti F. Macaluso A. Di Russo F.. (2014). Alpha band cortico-muscular coherence occurs in healthy individuals during mechanically-induced tremor. PLoS ONE 9:e115012. 10.1371/journal.pone.0115012 25514444 PMC4267728

[B14] Cao C. Li D. Jiang T. Ince N. F. Zhan S. Zhang J.. (2015). Resting state cortical oscillations of patients with Parkinson disease and with and without subthalamic deep brain stimulation: a magnetoencephalography study. J. Clin. Neurophysiol. 32, 109–118. 10.1097/WNP.0000000000000137 25233246

[B15] Chakarov V. Naranjo J. R. Schulte-Mönting J. Omlor W. Huethe F. Kristeva R. (2009). Beta-range EEG-EMG coherence with isometric compensation for increasing modulated low-level forces. J. Neurophysiol. 102, 1115–1120. 10.1152/jn.91095.2008 19458142

[B16] Cilia R. Landi A. Vergani F. Sganzerla E. Pezzoli G. Antonini A. (2007). Extradural motor cortex stimulation in Parkinson's disease. Mov. Disord. 22, 111–114. 10.1002/mds.21207 17083104

[B17] Cooper S. E. McIntyre C. C. Fernandez H. H. Vitek J. L. (2013). Association of deep brain stimulation washout effects with Parkinson disease duration. JAMA Neurol. 70, 95–99. 10.1001/jamaneurol.2013.581 23070397 PMC5148628

[B18] Crone N. E. Miglioretti D. L. Gordon B. Lesser R. P. (1998a). Functional mapping of human sensorimotor cortex with electrocorticographic spectral analysis. II. Event-related synchronization in the gamma band. Brain J. Neurol. 121(Pt 12), 2301–2315. 10.1093/brain/121.12.2301 9874481

[B19] Crone N. E. Miglioretti D. L. Gordon B. Sieracki J. M. Wilson M. T. Uematsu S. (1998b). Functional mapping of human sensorimotor cortex with electrocorticographic spectral analysis. I. Alpha and beta event-related desynchronization. Brain J. Neurol. 121(Pt 12), 2271–2299. 10.1093/brain/121.12.2271 9874480

[B20] de Hemptinne C. Swann N. C. Ostrem J. L. Ryapolova-Webb E. S. San Luciano M. Galifianakis N. B.. (2015). Therapeutic deep brain stimulation reduces cortical phase-amplitude coupling in Parkinson's disease. Nat. Neurosci. 18, 779–786. 10.1038/nn.3997 25867121 PMC4414895

[B21] Deuschl G. Schade-Brittinger C. Krack P. Volkmann J. Schäfer H. Bötzel K.. (2006). A randomized trial of deep-brain stimulation for Parkinson's disease. N. Engl. J. Med. 355, 896–908. 10.1056/NEJMoa060281 16943402

[B22] Devos D. (2004). Subthalamic nucleus stimulation modulates motor cortex oscillatory activity in Parkinson's disease. Brain 127, 408–419. 10.1093/brain/awh053 14691060

[B23] Engel A. K. Fries P. (2010). Beta-band oscillations–signalling the status quo? Curr. Opin. Neurobiol. 20, 156–165. 10.1016/j.conb.2010.02.015 20359884

[B24] Eusebio A. Cagnan H. Brown P. (2012). Does suppression of oscillatory synchronisation mediate some of the therapeutic effects of DBS in patients with Parkinson's disease? Front. Integr. Neurosci. 6:47. 10.3389/fnint.2012.00047 22787444 PMC3392592

[B25] Eusebio A. Thevathasan W. Doyle Gaynor L. Pogosyan A. Bye E. Foltynie T.. (2011). Deep brain stimulation can suppress pathological synchronisation in parkinsonian patients. J. Neurol. Neurosurg. Psychiatr. 82, 569–573. 10.1136/jnnp.2010.217489 20935326 PMC3072048

[B26] Gradinaru V. Mogri M. Thompson K. R. Henderson J. M. Deisseroth K. (2009). Optical deconstruction of parkinsonian neural circuitry. Science 324, 354–359. 10.1126/science.1167093 19299587 PMC6744370

[B27] Gross J. Kujala J. Hamalainen M. Timmermann L. Schnitzler A. Salmelin R. (2001). Dynamic imaging of coherent sources: studying neural interactions in the human brain. Proc. Natl. Acad. Sci. U.S.A. 98, 694–699. 10.1073/pnas.98.2.694 11209067 PMC14650

[B28] Grosse-Wentrup M. Schölkopf B. Hill J. (2011). Causal influence of gamma oscillations on the sensorimotor rhythm. Neuroimage 56, 837–842. 10.1016/j.neuroimage.2010.04.265 20451626

[B29] Hartmann C. J. Chaturvedi A. Lujan J. L. (2015). Quantitative analysis of axonal fiber activation evoked by deep brain stimulation via activation density heat maps. Front. Neurosci. 9:28. 10.3389/fnins.2015.00028 25713510 PMC4322637

[B30] Hillebrand A. Fazio P. de Munck J. C. van Dijk B. W. (2013). Feasibility of clinical Magnetoencephalography (MEG) functional mapping in the presence of dental artefacts. Clin. Neurophysiol. 124, 107–113. 10.1016/j.clinph.2012.06.013 22832101

[B31] Hipp J. F. Engel A. K. Siegel M. (2011). Oscillatory synchronization in large-scale cortical networks predicts perception. Neuron 69, 387–396. 10.1016/j.neuron.2010.12.027 21262474

[B32] Hirschmann J. Hartmann C. J. Butz M. Hoogenboom N. Ozkurt T. E. Elben S.. (2013a). A direct relationship between oscillatory subthalamic nucleus-cortex coupling and rest tremor in Parkinson's disease. Brain J. Neurol. 136, 3659–3670. 10.1093/brain/awt271 24154618

[B33] Hirschmann J. Özkurt T. E. Butz M. Homburger M. Elben S. Hartmann C. J.. (2011). Distinct oscillatory STN-cortical loops revealed by simultaneous MEG and local field potential recordings in patients with Parkinson's disease. Neuroimage 55, 1159–1168. 10.1016/j.neuroimage.2010.11.063 21122819

[B34] Hirschmann J. Özkurt T. E. Butz M. Homburger M. Elben S. Hartmann C. J.. (2013b). Differential modulation of STN-cortical and cortico-muscular coherence by movement and levodopa in Parkinson's disease. Neuroimage 68, 203–213. 10.1016/j.neuroimage.2012.11.036 23247184

[B35] Kelly P. J. (1996). The interactive use of magnetoencephalography in stereo- tactic image-guided neurosurgery. Neurosurgery 39, 92–102. 10.1097/00006123-199607000-00018 8805144

[B36] Kilner J. M. Baker S. N. Salenius S. Hari R. Lemon R. N. (2000). Human cortical muscle coherence is directly related to specific motor parameters. J. Neurosci. Off. J. Soc. Neurosci. 20, 8838–8845. 11102492 10.1523/JNEUROSCI.20-23-08838.2000 PMC6773054

[B37] Knowlton R. C. Shih J. (2004). Magnetoencephalography in epilepsy. Epilepsia 45(Suppl. 4), 61–71. 10.1111/j.0013-9580.2004.04012.x 15281961

[B38] Kringelbach M. L. Green A. L. Owen S. L. F. Schweder P. M. Aziz T. Z. (2010). Sing the mind electric - principles of deep brain stimulation. Eur. J. Neurosci. 32, 1070–1079. 10.1111/j.1460-9568.2010.07419.x 21039946

[B39] Kühn A. A. (2004). Event-related beta desynchronization in human subthalamic nucleus correlates with motor performance. Brain 127, 735–746. 10.1093/brain/awh106 14960502

[B40] Kühn A. A. Kempf F. Brücke C. Gaynor Doyle L. Martinez-Torres I. Pogosyan A.. (2008). High-frequency stimulation of the subthalamic nucleus suppresses oscillatory beta activity in patients with Parkinson's disease in parallel with improvement in motor performance. J. Neurosci. Off. J. Soc. Neurosci. 28, 6165–6173. 10.1523/JNEUROSCI.0282-08.2008 18550758 PMC6670522

[B41] Kühn A. A. Kupsch A. Schneider G. H. Brown P. (2006). Reduction in subthalamic 8–35 Hz oscillatory activity correlates with clinical improvement in Parkinson's disease. Eur. J. Neurosci. 23, 1956–1960. 10.1111/j.1460-9568.2006.04717.x 16623853

[B42] Li Q. Ke Y. Chan D. C. W. Qian Z.-M. Yung K. K. L. Ko H.. (2012). Therapeutic deep brain stimulation in Parkinsonian rats directly influences motor cortex. Neuron 76, 1030–1041. 10.1016/j.neuron.2012.09.032 23217750

[B43] Little S. Brown P. (2012). What brain signals are suitable for feedback control of deep brain stimulation in Parkinson's disease? Ann. N.Y. Acad. Sci. 1265, 9–24. 10.1111/j.1749-6632.2012.06650.x 22830645 PMC3495297

[B44] Little S. Brown P. (2014). The functional role of beta oscillations in Parkinson's disease. Parkinsonism Relat. Disord. 20(Suppl.) 1, S44–S48. 10.1016/S1353-8020(13)70013-0 24262186

[B45] Litvak V. Eusebio A. Jha A. Oostenveld R. Barnes G. R. Penny W. D.. (2010). Optimized beamforming for simultaneous MEG and intracranial local field potential recordings in deep brain stimulation patients. Neuroimage 50, 1578–1588. 10.1016/j.neuroimage.2009.12.115 20056156 PMC3221048

[B46] Litvak V. Jha A. Eusebio A. Oostenveld R. Foltynie T. Limousin P.. (2011). Resting oscillatory cortico-subthalamic connectivity in patients with Parkinson's disease. Brain J. Neurol. 134, 359–374. 10.1093/brain/awq332 21147836

[B47] Mäkelä J. Taulu S. Pohjola J. Ahonen A. Pekkonen E. (2007). Effects of subthalamic nucleus stimulation on spontaneous sensorimotor MEG activity in a Parkinsonian patient. Int. Congr. Ser. 1300, 345–348. 10.1016/j.ics.2007.02.003

[B48] Maris E. Oostenveld R. (2007). Nonparametric statistical testing of EEG- and MEG-data. J. Neurosci. Methods 164, 177–190. 10.1016/j.jneumeth.2007.03.024 17517438

[B49] Marreiros A. C. Cagnan H. Moran R. J. Friston K. J. Brown P. (2013). Basal ganglia-cortical interactions in Parkinsonian patients. Neuroimage 66, 301–310. 10.1016/j.neuroimage.2012.10.088 23153964 PMC3573233

[B50] Marsden J. Limousin-Dowsey P. Fraix V. Pollak P. Odin P. Brown P. (2001). Intermuscular coherence in Parkinson's disease: effects of subthalamic nucleus stimulation. Neuroreport 12, 1113–1117. 10.1097/00001756-200105080-00013 11338175

[B51] McIntyre C. C. Hahn P. J. (2010). Network perspectives on the mechanisms of deep brain stimulation. Neurobiol. Dis. 38, 329–337. 10.1016/j.nbd.2009.09.022 19804831 PMC2862840

[B52] Medvedovsky M. Taulu S. Bikmullina R. Ahonen A. Paetau R. (2009). Fine tuning the correlation limit of spatio-temporal signal space separation for magnetoencephalography. J. Neurosci. Methods 177, 203–211. 10.1016/j.jneumeth.2008.09.035 18996412

[B53] Mitra P. P. Pesaran B. (1999). Analysis of Dynamic Brain Imaging Data. Biophys. J. 76, 691–708. 10.1016/S0006-3495(99)77236-X 9929474 PMC1300074

[B54] Moro E. Schwalb J. M. Piboolnurak P. Poon Y. Y. W. Hamani C. Hung S. W. (2011). Unilateral subdural motor cortex stimulation improves essential tremor but not Parkinson's disease. Brain, 134, 2096–2105. 10.1093/brain/awr072 21646329

[B55] Myers L. J. Lowery M. O'Malley M. Vaughan C. L. Heneghan C. St Clair Gibson A.. (2003). Rectification and non-linear pre-processing of EMG signals for cortico-muscular analysis. J. Neurosci. Methods 124, 157–165. 10.1016/S0165-0270(03)00004-9 12706845

[B56] Oostenveld R. Fries P. Maris E. Schoffelen J.-M. (2011). FieldTrip: open source software for advanced analysis of MEG, EEG, and invasive electrophysiological data. Comput. Intell. Neurosci. 2011, 1–9. 10.1155/2011/156869 21253357 PMC3021840

[B57] Orrison W. W. Jr. (1999). Magnetic source imaging in stereotactic and functional neurosurgery. Stereotact. Funct. Neurosurg. 72, 89–94. 10.1159/000029705 10928916

[B58] Pizzella V. Romani G. (1990). Principles of magnetoencephalography, in Magnetoencephalography, ed Sato S. (New York, NY: Raven Press), 1–9. 2270795

[B59] Rezai A. R. Hund M. Kronberg E. Zonenshayn M. Cappell J. Ribary U.. (1996). The interactive use of magnetoencephalography in stereotactic image-guided neurosurgery. Neurosurgery 39, 92–102. 10.1097/00006123-199607000-00018 8805144

[B60] Rosin B. Slovik M. Mitelman R. Rivlin-Etzion M. Haber S. N. Israel Z.. (2011). Closed-loop deep brain stimulation is superior in ameliorating parkinsonism. Neuron 72, 370–384. 10.1016/j.neuron.2011.08.023 22017994

[B61] Salenius S. Avikainen S. Kaakkola S. Hari R. Brown P. (2002). Defective cortical drive to muscle in Parkinson's disease and its improvement with levodopa. Brain, J. Neurol. 125, 491–500. 10.1093/brain/awf042 11872607

[B62] Schoffelen J.-M. Oostenveld R. Fries P. (2005). Neuronal coherence as a mechanism of effective corticospinal interaction. Science 308, 111–113. 10.1126/science.1107027 15802603

[B63] Schuepbach W. M. M. Rau J. Knudsen K. Volkmann J. Krack P. Timmermann L.. (2013). Neurostimulation for Parkinson's disease with early motor complications. N. Engl. J. Med. 368, 610–622. 10.1056/NEJMoa1205158 23406026

[B64] Selzler K. Burack M. Bender R. Mapstone M. (2013). Neurophysiological correlates of motor and working memory performance following subthalamic nucleus stimulation. J. Cogn. Neurosci. 25, 37–48. 10.1162/jocn_a_00306 23198889

[B65] Silberstein P. Pogosyan A. Kühn A. A. Hotton G. Tisch S. Kupsch A.. (2005). Cortico-cortical coupling in Parkinson's disease and its modulation by therapy. Brain J. Neurol. 128, 1277–1291. 10.1093/brain/awh480 15774503

[B66] Swann N. C. de Hemptinne C. Aron A. R. Ostrem J. L. Knight R. T. Starr P. A. (2015). Elevated synchrony in Parkinson disease detected with electroencephalography. Ann. Neurol. 78, 742–750. 10.1002/ana.24507 26290353 PMC4623949

[B67] Taulu S. Hari R. (2009). Removal of magnetoencephalographic artifacts with temporal signal-space separation: demonstration with single-trial auditory-evoked responses. Hum. Brain Mapp. 30, 1524–1534. 10.1002/hbm.20627 18661502 PMC6871056

[B68] Taulu S. Simola J. (2006). Spatiotemporal signal space separation method for rejecting nearby interference in MEG measurements. Phys. Med. Biol. 51, 1759–1768. 10.1088/0031-9155/51/7/008 16552102

[B69] Timmermann L. Gross J. Dirks M. Volkmann J. Freund H.-J. Schnitzler A. (2003). The cerebral oscillatory network of parkinsonian resting tremor. Brain 126, 199–212. 10.1093/brain/awg022 12477707

[B70] Tropini G. Chiang J. Wang Z. J. Ty E. McKeown M. J. (2011). Altered directional connectivity in Parkinson's disease during performance of a visually guided task. Neuroimage 56, 2144–2156. 10.1016/j.neuroimage.2011.03.018 21402160

[B71] Weiss D. Breit S. Hoppe J. Hauser A.-K. Freudenstein D. Krüger R.. (2012). Subthalamic nucleus stimulation restores the efferent cortical drive to muscle in parallel to functional motor improvement. Eur. J. Neurosci. 35, 896–908. 10.1111/j.1460-9568.2012.08014.x 22393899

[B72] Weiss D. Klotz R. Govindan R. B. Scholten M. Naros G. Ramos-Murguialday A. (2015). Subthalamic stimulation modulates cortical motor network activity and synchronization in Parkinson's disease. Brain 138(Pt 3), 679–693. 10.1093/brain/awu380 25558877 PMC4408429

[B73] Weiss D. Walach M. Meisner C. Fritz M. Scholten M. Breit S.. (2013). Nigral stimulation for resistant axial motor impairment in Parkinson's disease? A randomized controlled trial. Brain J. Neurol. 136, 2098–2108. 10.1093/brain/awt122 23757762 PMC3692032

[B74] Whitmer D. de Solages C. Hill B. Yu H. Henderson J. M. Bronte-Stewart H. (2012). High frequency deep brain stimulation attenuates subthalamic and cortical rhythms in Parkinson's disease. Front. Hum. Neurosci. 6:155. 10.3389/fnhum.2012.00155 22675296 PMC3366347

[B75] Yao B. Salenius S. Yue G. H. Brown R. W. Liu J. Z. (2007). Effects of surface EMG rectification on power and coherence analyses: an EEG and MEG study. J. Neurosci. Methods 159, 215–223. 10.1016/j.jneumeth.2006.07.008 16949676

